# *Toxoplasma* Controls Host Cyclin E Expression through the Use of a Novel MYR1-Dependent Effector Protein, HCE1

**DOI:** 10.1128/mBio.00674-19

**Published:** 2019-04-30

**Authors:** Michael W. Panas, Adit Naor, Alicja M. Cygan, John C. Boothroyd

**Affiliations:** a Department of Microbiology and Immunology, Stanford School of Medicine, Stanford, California, USA; Albert Einstein College of Medicine

**Keywords:** effector proteins, *Toxoplasma*, host-pathogen interactions, microbiology

## Abstract

Toxoplasma gondii is an obligate intracellular parasite that establishes a favorable environment in the host cells in which it replicates. We have previously reported that it uses MYR-dependent translocation of dense granule proteins to elicit a key set of host responses related to the cell cycle, specifically, E2F transcription factor targets, including cyclin E. We report here the identification of a novel *Toxoplasma* effector protein that is exported from the parasitophorous vacuole in a MYR1-dependent manner and localizes to the host’s nucleus. Parasites lacking this inducer of host cyclin E (HCE1) are unable to modulate E2F transcription factor target genes and exhibit a substantial growth defect. Immunoprecipitation of HCE1 from infected host cells showed that HCE1 efficiently binds elements of the cyclin E regulatory complex, namely, DP1 and its partners E2F3 and E2F4. Expression of HCE1 in Neospora caninum, or in uninfected human foreskin fibroblasts (HFFs), showed localization of the expressed protein to the host nuclei and strong cyclin E upregulation. Thus, HCE1 is a novel effector protein that is necessary and sufficient to impact the E2F axis of transcription, resulting in co-opting of host functions to the advantage of *Toxoplasma*.

## Abstract (precis)

Like most Apicomplexan parasites, Toxoplasma gondii has the remarkable ability to invade and establish a replicative niche within another eukaryotic cell, in this case, any of a large number of cell types in almost any warm-blooded animals. Part of the process of establishing this niche is the export of effector proteins to co-opt host cell functions in favor of the parasite. Here we identify a novel effector protein, HCE1, that the parasites export into the nucleus of human cells, where it modulates the expression of multiple genes, including the gene encoding cyclin E, one of the most crucial proteins involved in controlling when and whether a human cell divides. We show that HCE1 works through binding to specific transcription factors, namely, E2F3, E2F4, and DP1, that normally carefully regulate these all-important pathways. This represents a new way in which these consummately efficient infectious agents co-opt the human cells that they so efficiently grow within.

## INTRODUCTION

Intracellular infectious agents face unique challenges and opportunities. One such is interfacing with the host cell cycle, and many have evolved ways to speed up, slow down, or otherwise disrupt this process. Toxoplasma gondii is an obligate intracellular eukaryote that conforms to this rule. Indeed, this ubiquitous member of the phylum *Apicomplexa* has previously been described to cause host cells to stall in states ranging between the S phase and the G_2_/M transition (1). In some host cells, this manifests in the host cell endocycling and duplicating its DNA without subsequent cytokinesis, and previously reported evidence has suggested that this is mediated by an active but unknown parasite-derived activity (2).

*Toxoplasma* tachyzoites use secreted effectors derived from the dense granules to manipulate host cell functions while replicating in the parasitophorous vacuole (PV) (3, 4). A select set of these dense granule proteins can cross the parasitophorous vacuole membrane (PVM) and enter the host cytoplasm (5) in a process that is dependent on at least four parasite proteins; three of these are located at the PVM and have been termed MYR1, MYR2, and MYR3 (2, 6), whereas a fourth, aspartyl protease 5 (ASP5), is found within the Golgi and catalyzes proteolysis at a conserved Arg-Arg-Leu (RRL) sequence (7, 8). To date, GRA16 (6, 9), GRA24 (2), and GRA18 (10) have been shown to employ this machinery and the loss of the T.gondii IST (TgIST)-induced phenotype in Myr^–^ mutants is consistent with it being a fourth such protein (11).

Using mutants defective in MYR1 and ASP5 and using human foreskin fibroblasts (HFFs) as the host cell, we recently described the totality of impacts on the host transcriptome that are dependent on effectors that use this system to translocate across the PVM. The data showed the expected impacts on host processes already known to be caused by GRA16, GRA24, GRA18, and TgIST. They also showed, however, a profound and unexplained MYR1-dependent impact on gene sets defined as E2F target genes and/or G_2_/M checkpoint control genes (12). The E2F transcription factors are a family of DNA binding proteins that form a heterodimer with “dimerization partner” 1 (DP1) and regulate a cohort of genes, including the genes encoding cyclin E and its cyclin-dependent kinase (CDK2), which control the progression of the cell cycle (13).

To identify the effector responsible for these E2F-mediated effects, we took a bioinformatic approach, focusing on candidate proteins that would be predicted to traffic across the PVM, reach the host nucleus, and impact the host cell cycle. This approach proved successful, and we describe here a novel *Toxoplasma* protein whose entry into the host cell is MYR1 dependent, that binds to E2F/DP1 heterodimers, and that is both necessary and sufficient for upregulation of host cyclin E, a key regulator of the host cell cycle.

## RESULTS

### TGGT1_239010 is a dense granule protein that traffics to the host nucleus in a MYR1- and ASP5-dependent manner.

Similarities exist among the four known effector proteins that transit across the PVM, and we sought to harness this information to identify the effector that might be mediating the upregulation of cyclin E. Specifically, the known effectors all originate in the dense granules, are exported from the parasite, and do not end up inserted in a membrane but rather transit to the host cytosol or nucleus, where many then drive a function that is specific to cells infected with *Toxoplasma* compared to those infected with a species of a closely related genus, Neospora caninum. The latter point was specifically true in the case of cyclin E, where transcriptomic analyses have shown that *Toxoplasma* upregulates this gene strongly whereas the data for *Neospora* revealed no such impact (14).

On the basis of all this, we searched existing excreted secreted antigen lists (15) for proteins predicted to have (i) a signal sequence for export; (ii) no transmembrane domain that might prevent translocation across the PVM; (iii) a nuclear localization signal (NLS) to mediate import into the host nucleus; and (iv) either no orthologue in Neospora caninum or a very low level of similarity with such a gene. For the first two criteria, we used the predictions in ToxoDB (version 27, released 19 February 2016) and then confirmed these with the publicly available prediction software Phobius (http://phobius.sbc.su.se/), a combined transmembrane topology and signal peptide predictor.

**FIG 1:**
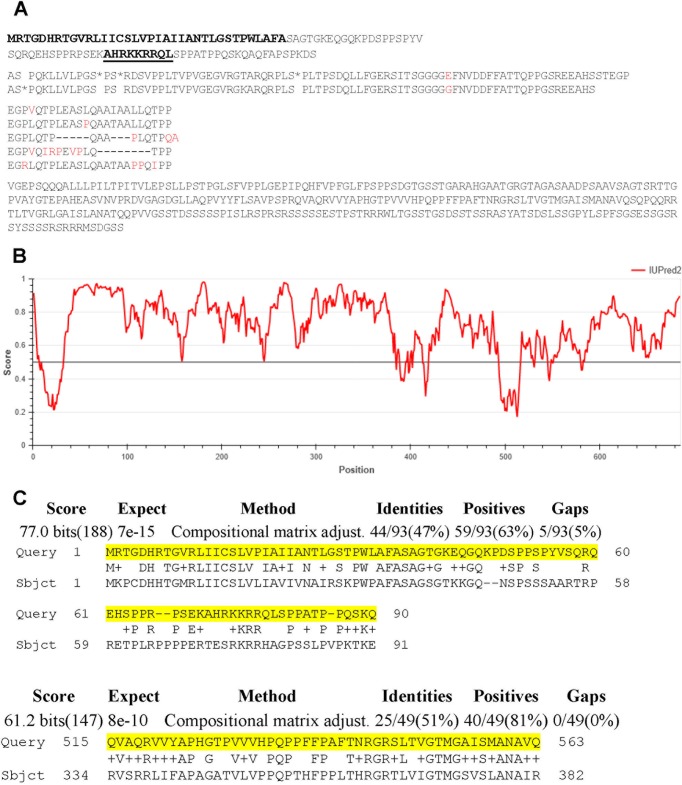
Predicted amino acid sequence and homology of TGGT1_239010. (A) *TGGT1_239010* codes for a 685-amino-acid protein that contains a predicted signal peptide (bold lettering) but no transmembrane domains internal to the protein, consistent with predictions for other effectors originating in dense granules. There are also several repeated domains of unknown function but repetitive structure and a predicted nuclear localization signal (underlined and highlighted in boldface). Red letters indicate discordance between the repeats, and asterisks (*) indicate serine residues previously identified as being phosphorylated after secretion. Spaces have been added to make the repeated domains clearer. (B) Disorder of TgGT1_239010 as predicted by the program IUPred2A (https://iupred2a.elte.hu/). IUPred2A predicts global structural disorder encompassing at least 30 consecutive residues of the protein and returns a score between 0 and 1 for each residue, corresponding to the probability of the given residue being part of a disordered region. (C) TGGT1_239010 is highly dissimilar to its orthologue BN1204_015825 in Neospora caninum. NCBI BLAST was used to compare TGGT1_239010 (Query) to the *Neospora* proteome, and the only similarities found were within the two displayed regions of BN1204_015825 (Sbjct), including the predicted signal peptide and a segment of ∼50 amino acids toward the C terminus. The numbering indicates the amino acid position relative to the N terminus in each predicted protein.

One gene that met these criteria was *TGGT1_239010*, which is predicted to encode a 685-amino-acid protein with a signal sequence and no transmembrane domains (Fig. 1A). Previously published phosphoproteomic data examining differential phosphorylation states of proteins in parasites within intact vacuoles versus syringe-released parasites give insight into whether these posttranslational modifications are done inside the parasite or within the host/PV. While this data set cannot distinguish between host and PV, it indicates that TGGT1_239010 is phosphorylated at 4 serines after being secreted from the parasite (ToxoDB [16]), consistent with phosphorylation of other effector proteins. TGGT1_239010 also contains a predicted monopartite NLS downstream of the signal sequence that was identified by the use of NLS mapper software (http://nls-mapper.iab.keio.ac.jp/cgi-bin/NLS_Mapper_form.cgi) as AHRKKRRQL with a score of 8.5, suggesting a high likelihood of its being a eukaryotic NLS (a score of 7 represents the threshold for sole localization to the nucleus). Following the NLS, there is a run of 87 amino acids that is nearly perfectly duplicated once in the type I GT1 and type III VEG strains and twice in the type II ME49 strain (https://toxodb.org/toxo/). Following the large repeated domain, there is an approximately 24-amino-acid sequence that is imperfectly repeated five times before the sequence ends in a predicted disordered C terminus (Fig. 1B) (based on data from https://iupred2a.elte.hu/). These repeated sequences are consistent with conserved domains observed in other effector proteins, such as GRA16 and GRA24, where they have been reported to play a role in binding their cognate interacting partners. Additionally, the presence of the unstructured, serine-rich C-terminal region is consistent with the hypothesis that unstructured regions act as dynamic regions able to accommodate more signaling partners, as well as allowing unfolding in order to cross the PVM (5). Thus, the DNA sequence of *TGGT1_239010* suggests that it encodes a protein that is exported, does not get stuck in a membrane, and would localize to the host nucleus.

NCBI BLASTP identified an orthologue, BN1204_015825, that is present in Neospora caninum but with only two short regions of significant similarity: 47% identity (63% similarity) over the first 90 amino acids, mostly at the extreme N-terminal region corresponding to the predicted signal peptide, and 51% identity (81% similarity) over a stretch of just 49 amino acids in the disordered C-terminal region (Fig. 1C). This argues that TGGT1_239010 undergoes strong positive selection and that the function of the *Neospora* orthologue is likely very different from that of TGGT1_239010.

**FIG 2:**
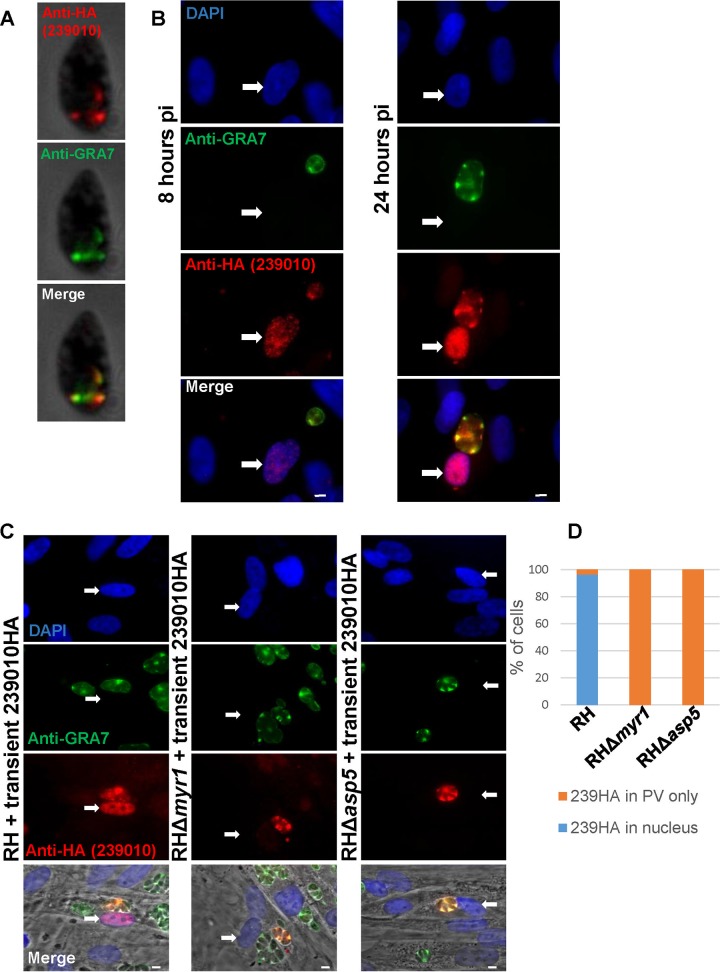
TGGT1_239010 is a dense granule protein that traffics to the nucleus of infected cells in a MYR1- and ASP5-dependent manner. (A) Immunofluorescence assay (IFA) using anti-HA antibodies (red) to stain extracellular RH tachyzoites expressing TGGT1_239010 with an HA tag controlled by its endogenous promoter, showing colocalization with known dense granule protein GRA7 (green) at discrete puncta within the parasite. The white scale bar represents 5 μm. (B) IFA was performed as described for panel A, but the images represent RH tachyzoites infecting human foreskin fibroblasts (HFFs) at 8 or 24 h postinfection (pi), with DAPI staining to show the host nuclei. The white arrow points to the host nucleus in the infected cell. (C) IFA was performed as described for panel B, but the images show representative HFFs infected for 20 h with wild-type (RH), RHΔ*myr1*, or RHΔ*asp5* parasites transiently transfected with a construct expressing HA-tagged TGGT1_239010. TGGT1_239010 traffics to the host nucleus in strain RH-infected HFFs but not in strain RHΔ*myr1*-infected HFFs or strain RHΔ*asp5*-infected HFFs. (D) Quantification of the IFA data from the experiments whose results are shown in panel C. The data corresponding to the *y* axis (labeled “% of cells”) represent percentages of infected host cells showing nuclear or PV staining with anti-HA antibody after infection with the indicated parasite line. Wild-type RH, *n* = 28; strain RHΔ*myr1*, *n* = 41; strain RHΔ*asp5*, *n* = 29. Data shown are representative of two independent experiments that gave similar results.

To begin characterization of *TGGT1_239010*, we first cloned its open reading frame, its 5′-untranslated region, and, to avoid overexpression artifacts, its predicted promoter into the plasmid pGRA and appended a sequence representing a C-terminal hemagglutinin (HA) tag. Extracellular tachyzoites expressing this construct, referred to as 239HA, were stained with antibodies for HA and for GRA7, as a well-characterized marker for dense granules. Colocalization in discrete puncta was generally but not universally observed (Fig. 2A), consistent with TGGT1_239010 being a dense granule protein or a member of the “dense granule-like” proteins that have been observed for GRA16/MYR1/TgIST (2, 9, 11).

When the same tachyzoites were used to infect human foreskin fibroblasts (HFFs) for 8 h, 239HA localized within the parasitophorous vacuole, again with strong overlapping of GRA7 (Fig. 2B). At 24 h postinfection (hpi), it was apparent from the colocalization with GRA7 that the 239HA within the PV was largely located in the spaces between the parasites. More importantly, at both 8 and 24 hpi, 239HA also localized to the infected cell’s host nucleus, demonstrating that it is exported from the vacuole and suggesting that the predicted NLS is functional. Comparing the results for 8 and 24 hpi, staining of 239HA increased over time in both the parasitophorous vacuole and nucleus, as previously noted for dense granule proteins (9).

All dense granule proteins known to localize to the host cell nucleus require the MYR machinery as well as an active ASP5 for translocation across the PVM, regardless of whether they are themselves cleaved by ASP5. To test if this was also the case for TGGT1_239010, we transiently transfected the wild-type RH strain (RH-WT) and mutant strains RHΔ*myr1* and RHΔ*asp5* with a plasmid expressing 239HA. Expression of the transgene was confirmed in all three strains based on anti-HA staining in the parasitophorous vacuole, colocalizing with GRA7 (Fig. 2C); however, there was observable 239HA in the host cell nucleus only in cells infected with the wild-type RH. Quantification of the results showed that 239HA was clearly present in the host nucleus in 96% of cells infected with the wild-type parasites, whereas no such nuclear staining was observed in the cells infected with the RHΔ*myr1* or RHΔ*asp5* mutants (Fig. 2D). Thus, as in the case of GRA24 (7), even though TGGT1_239010 has no TEXEL motif (Arg-Arg-Leu) and was not detected in an assay performed to identify all proteins cleaved by ASP5 (17), its export requires both this protease and intact MYR machinery.

### TGGT1_239010 is necessary and sufficient for host cyclin E induction.

To determine if TGGT1_239010 is the effector controlling cyclin E (CCNE1) expression, we next compared the host cell response observed during infection with wild-type parasites to the response seen during infection with parasites lacking TGGT1_239010. To create such a strain, we used clustered regularly interspaced short palindromic repeat (CRISPR) analysis to target the single-exon genomic sequence of *TGGT1_239010* for disruption by insertion of the *HXGPRT* gene (Fig. S1A). Following selection for insertion of *HXGPRT*, we isolated clones and used PCR to amplify the *TGGT1_239010* locus using primers flanking the targeted insertion site. The results (Fig. S1B) show the predicted change in the size of the PCR product, indicating disruption in the clones, and subsequent sequencing confirmed the disruptive insertion in *TGGT1_239010*.

10.1128/mBio.00674-19.1 FIG S1 Deletion of the single-exon *TGGT1_239010* gene by CRISPR-targeted insertion of *HXGPRT*. (A) Schematic of CRISPR-mediated disruption of *TGGT1_239010* followed by insertion of the *HXGPRT* gene for selection. Primers flanking the guide-targeted region, indicated by “Forward” and “Reverse,” were constructed to amplify a ∼500-bp region of the gene. (B) PCR amplification of DNA from wild-type RH and a HXGPRT^+^ strain with disruption of *TGGT1_239010* (RH*Δ239010*) using the forward and reverse primers shown in panel A. The size markers (M) reveal bands of the expected size in the RH strain (∼500 bp) and in the RH*Δ239010* strain (∼1,700 bp). Sequencing of the amplification products confirmed the identities of these bands as the result of PCR, as predicted from the schematic in panel A. Download FIG S1, DOCX file, 0.1 MB. Copyright © 2019 Panas et al. 2019 Panas et al. This content is distributed under the terms of the Creative Commons Attribution 4.0 International license.

**FIG 3:**
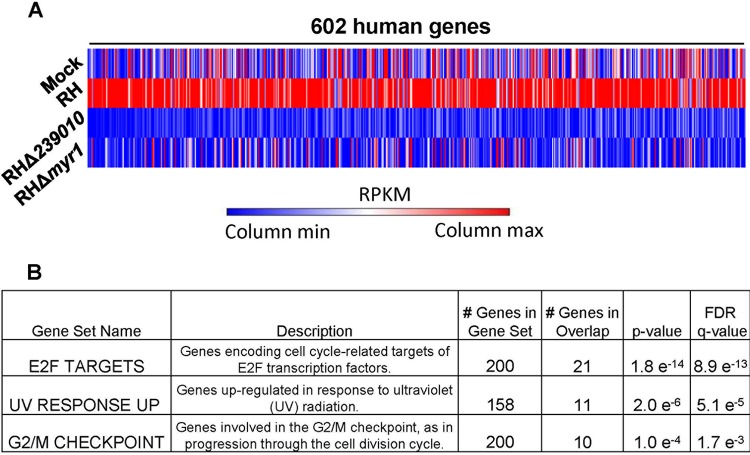
Disruption of Toxoplasma *TGGT1_239010* results in a parasite unable to alter the expression of genes associated with the host cell cycle. (A) Graphical representation of results of RNA-Seq analysis of HFFs subjected to mock infection or infected with the indicated *Toxoplasma* strains 6 h postinfection. Values shown represent average relative RPKM values from two independent experiments, with red and blue indicating the highest and lowest values, respectively, for each gene. Shown are the 602 human genes that were annotated and that showed a minimum of 2.5-fold upregulation in cells infected with the wild-type RH strain versus strain RHΔ*239010*. The values for these same genes in mock-infected HFFs and in cells infected with strain RHΔ*myr1* are shown for comparison. Gene names and RPKM values can be found in Table S1. (B) Gene set enrichment analysis for the collection of host genes that are differentially upregulated (*P* value < 0.0001) in HFFs infected with strain RHΔ*239010* versus the wild-type RH strain. Specific genes in each gene set are listed in Table S2.

With a confirmed knockout in hand, we next performed transcriptome sequencing (RNA-Seq) on mock-infected HFFs and HFFs infected for 6 h with wild-type RH, strain RHΔ*239010*, and strain RHΔ*myr1* at a high multiplicity of infection (MOI) of 5. We chose this MOI to be sure the vast majority of the HFFS were infected and the 6-h time point to allow parasites sufficient time to invade, establish a parasitophorous vacuole, and export TGGT1_239010, thereby altering host functions. We reasoned this would minimize the amount of downstream effects, with the goal of catching mostly the primary host targets of TGGT1_239010, should it prove to be the effector we sought. The results (Fig. 3A) showed that deletion of *TGGT1_23901*0 has a major impact on the ability of *Toxoplasma* to modulate host cell transcription. Using a threshold of at least a 2.5-fold difference in a host gene’s transcript levels, we observed that 602 annotated genes were upregulated in cells infected with wild-type RH that were not upregulated in cells infected with strain RHΔ*239010* (Table S1). The relative expression levels of these genes are also shown for HFF infected with strain RHΔ*myr1* and for those that were subjected to mock infection.

10.1128/mBio.00674-19.4 TABLE S1 RNA-Seq results for the 602 annotated genes shown in Fig. 3 that were expressed in RH-infected HFFs at levels that were least 2.5-fold higher than were seen in strain RHΔ*239010*-infected HFFs. RPKM values corresponding to the results of mock infection and infection with strain RH, strain RHΔ*239010*, and strain RHΔ*myr1* are indicated. Genes are ranked in decreasing order of the ratio of expression in strain RH versus expression in strain RHΔ*239010*. For instances in which there was no expression of the gene in RHΔ*239010*-infected cells, the genes with highest level of expression in RH-infected cells are listed first. Download Table S1, XLSX file, 0.05 MB. Copyright © 2019 Panas et al. 2019 Panas et al. This content is distributed under the terms of the Creative Commons Attribution 4.0 International license.

10.1128/mBio.00674-19.5 TABLE S2 A list of the host genes that were upregulated in RH-infected HFFs but not those infected with RHΔ*239010* parasites and that contributed to the three GSEA gene sets that were significantly enriched in the RH-infected cells versus RHΔ239010-infected cells, as shown in Fig. 3. Download Table S2, XLSX file, 0.01 MB. Copyright © 2019 Panas et al. 2019 Panas et al. This content is distributed under the terms of the Creative Commons Attribution 4.0 International license.

Gene set enrichment analysis (GSEA) was then applied to the set of genes that were differentially expressed between the strain RH-infected and strain RHΔ*239010-*infected HFFs, using a *P* value threshold of 1 × 10^−4^ to identify any gene sets that were significantly affected. The results (Fig. 3B) showed that the affected genes were most significantly enriched for genes impacted by the E2F family of transcription factors, having a *P* value of 1.8 × 10^−14^. We also observed significant effects on the “UV radiation response” gene set as well as on the “G_2_/M checkpoint” gene set, albeit at lower *P* values of 2.0 × 10^−6^ and 1.0 × 10^−4^, respectively. E2F transcription factors are generally known to initiate the G_1_/S transition by an upregulation of cyclin E (among other proteins), while UV response and G_2_/M checkpoint genes are involved in cell cycle checkpoints associated with both DNA replication and proceeding to mitosis. In total, GSEA (http://software.broadinstitute.org/gsea/index.jsp) identified 21 genes that were targeted by E2F molecules, many of which are well known as parts of the prereplication complex initiating DNA synthesis as follows: MCM2, MCM3, MCM4, MCM5, and MCM7 as well as the DNA polymerase POLE (Table S2).

Given the limited number of gene sets that appeared to be affected by the deletion of *TGGT1_239010*, as well as the fact that it was ultimately found in the host cell nucleus, it seemed unlikely that this protein was part of the effector translocation machinery, rather than being an effector itself. Nevertheless, and to confirm this, we tested whether the export of a different, known effector molecule, GRA16, was impacted by the loss of the *TGGT1_239010* gene. The results (Fig. S2) showed that GRA16 accumulates normally in the nucleus of cells infected with strain RHΔ*239010* when transiently transfected with an HA-tagged GRA16. Thus, TGGT1_239010 is not a part of the general translocation machinery.

10.1128/mBio.00674-19.2 FIG S2 Disruption of *TGGT1_239010* does not prevent GRA16 from exiting the parasitophorous vacuole and accessing the host cell nucleus. The RHΔ*239010* strain was transiently transfected with plasmid pGRA-GRA16HA, and GRA16HA localization (green) was assessed by IFA. GRA7 (red) was used to visualize the parasitophorous vacuoles, whether they harbored transfected parasites or not. The white scale bar represents 5 μm. Download FIG S2, DOCX file, 0.1 MB. Copyright © 2019 Panas et al. 2019 Panas et al. This content is distributed under the terms of the Creative Commons Attribution 4.0 International license.

**FIG 4:**
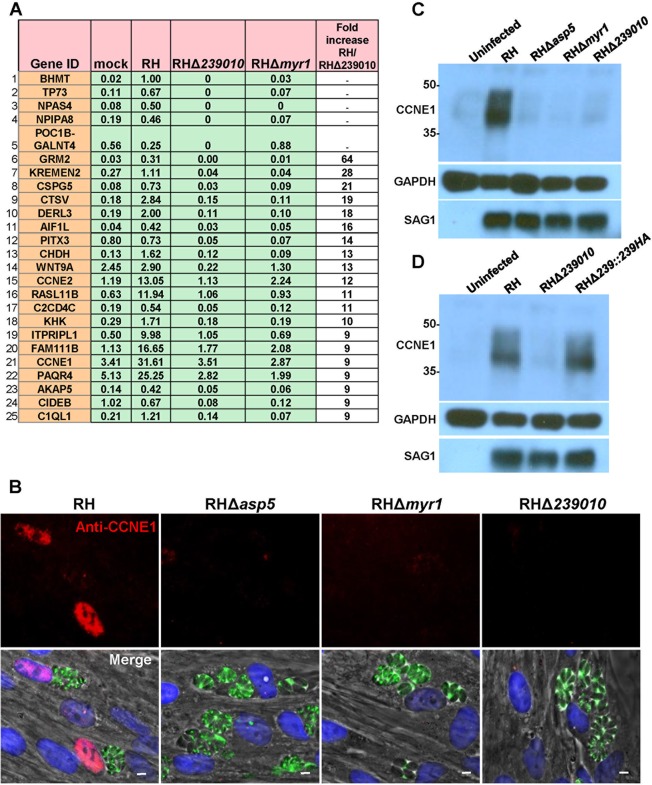
The cyclin E1 (*CCNE1*) and cyclin E2 (*CCNE2*) genes are among the top annotated genes whose upregulation is dependent on TGGT1_239010. (A) RPKM values for the top 25 human genes that had a minimum of 10 reads in both of the RH-infected samples and whose upregulation is most highly dependent on TGGT1_239010. Genes were sorted based on the ratio of their expression during wild type infection to that during infection with strain RHΔ*239010*. RPKM values for cells infected with strain RHΔ*myr1* are shown for comparison. For instances where there was no expression of the gene in strain RHΔ*239010*-infected cells, the genes with highest expression in RH-infected cells are listed first. (B) IFA was used to assess the expression of host cyclin E1 (red) in the nuclei (blue) of cells infected with the indicated strains (green) for 20 h. The white scale bar represents 5 μm. (C) Western blot of cyclin E1 expression from HFFs infected for 20 h with the indicated strains of *Toxoplasma*. Results from the same blots probed with antibodies for host GAPDH and parasite SAG1 are shown as loading controls. Size markers (kDa) are shown to the left. The blot shown is representative of results of three independent replicates. (D) The experiment was performed as described for the Western blots shown in panel C but with the addition of lysate from HFFs infected with a RHΔ*239010* strain carrying an ectopically located copy of the wild-type *TGGT1_239010* gene, including a sequence encoding a C-terminal HA tag (RHΔ*239010*::*239010-*HA). The blot shown is representative of three independent replicates.

To gain further information about the role of TGGT1_239010, we next ranked host genes by their fold differences in expression between RH-WT-infected and strain RHΔ*239010*-infected HFFs and examined those that had at least 10 reads per gene in the RH-infected sample, a number large enough for confidence in differential expression (Fig. 4A). Because E2F transcription factors drive cyclin E expression, it was not surprising to find that both cyclin E1 (CCNE1) and cyclin E2 (CCNE2) were among the 25 most highly affected genes. We chose to focus on these cyclins because they are canonical downstream targets of the E2F transcription factors and they play crucial roles in cell cycle control. To validate and extend the RNA-Seq results, we examined the protein levels of cyclin E1 in host cells infected with wild-type and strain RHΔ*239010* parasites. Consistent with the elevated expression of mRNA levels, an immunofluorescence assay (IFA) revealed that infected HFFs showed an upregulation of cyclin E1 expression dependent on the parasite being wild type for both MYR1 and ASP5 as well as on its having an intact *TGGT1_239010* gene (Fig. 4B). This observation was confirmed by Western blotting of infected cell lysates probed with antibodies to cyclin E1; cyclin E1 was strongly upregulated in HFFs infected with wild-type tachyzoites but not in HFFs infected with tachyzoites lacking MYR1, ASP5, or TGGT1_239010 (Fig. 4C). To ensure that the disruption of the *TGGT1_239010* locus was responsible for the phenotype, we generated a complemented strain (strain RHΔ*239010*::*239010HA*) and tested its ability to rescue the phenotype. The results (Fig. 4D) showed that, indeed, the complemented strain was fully capable of inducing the upregulation of cyclin E1 to levels indistinguishable from those seen in cells infected with wild-type RH. On the basis of this, we dubbed the *TGGT1_239010* locus the host cyclin E (HCE1) gene (*HCE1*) for its impact on “host cyclin E” expression.

### HCE1 is necessary for efficient parasite growth in HFFs.

**FIG 5:**
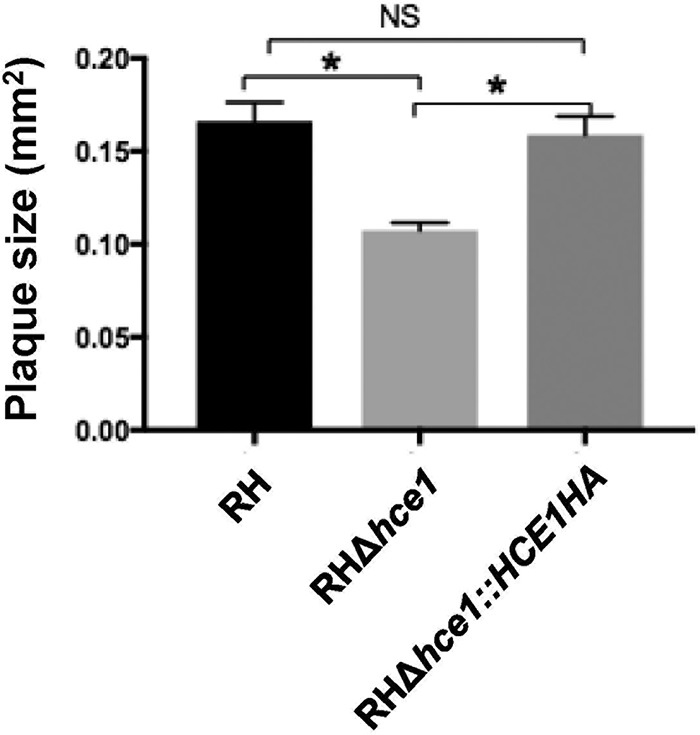
Growth of parasites in HFFs is retarded by the loss of HCE1. The indicated strains were used to infect HFFs, and the plaque size was measured after 7 days of growth. Average plaque size was assessed by ImageJ for *n* = 31 (strain RH), *n* = 38 (strain RHΔ*hce1*), and *n* = 30 (strain RHΔ*hce1*::*HCE1HA*) parasites. Data are representative of results from 3 independent biologic replicates, and error bars indicate standard errors of the means. Significance was determined by two-tailed *t* test. *, *P* < 0.0001; NS, not significant (*P* > 0.05).

To assess the importance of the function of HCE1 in parasite growth, we measured plaque area using wild-type and Δ*hce1* mutant parasites. The results (Fig. 5) show that the areas of plaques of tachyzoites lacking *HCE1* were ∼65% the areas of wild-type plaques at day 7 and that complementation with a copy of *HCE1HA* returned these plaques to their wild-type size. Thus, HCE1 confers a growth advantage *in vitro*, even in HFFs which lack many immune defenses, consistent with an impact on host cell cycle control rather than interfering with known immune responses.

### HCE1 binds host E2F/DP1 heterodimers that control host cell cycle.

To shed light on how HCE1 functions, we next sought to determine its binding partners. To do this, we used anti-HA antibodies to immunoprecipitate HA-tagged HCE1 from cells infected for 24 h with strain RHΔ*hce1*::*HCE1HA* tachyzoites under conditions where associating proteins were likely to remain intact (immunoprecipitation [IP] no. 1). As a control for proteins that might be precipitated with the anti-HA beads nonspecifically, we also performed this experiment with HFFs infected with an untagged RH strain. We performed this same experiment a second time under more-stringent conditions, disrupting weak interactions and releasing proteins from membranes with an additional sonication step and comparing the results to an untagged RH strain (IP no. 2). Mass spectrometry (MS) analysis of the proteins enriched by the anti-HA immunoprecipitation in both experiments was performed, and the results were ranked by the ratio of the relative numbers of spectral counts found for a given protein in the HCE1HA lysates compared to RH, with a nominal value of 1 added to all values to facilitate the mathematical calculation.

When we searched the IP-MS data for *Toxoplasma* proteins associating with HCE1, we observed that 11 were highly enriched (i.e., were assigned an enrichment score of ≥5 in both experiments) (Table S3). Among these were MYR1, which we know is necessary for HCE1 translocation, possibly explaining its association, and other GRA proteins that we know are secreted into the PV (GRA28 and GRA44), of which at least one (GRA28) is also translocated into the host cell (Table S3) (17, 18). One or more of these proteins may be an additional player(s) in the trafficking of HCE1 across the PVM and into the host nucleus, but we have not pursued this further because, as described further below, we found HCE1 can perform its major functions independently of all other *Toxoplasma* proteins.

10.1128/mBio.00674-19.6 TABLE S3 Mass spectrometry analysis parameters and results for proteins that coimmunoprecipitate with HCE1HA-expressing and untagged RH parasites. For all sheets, the IDs corresponding to the majority proteins, i.e., the proteins which contained at least half of the peptides belonging to a protein group (grouping of proteins which cannot be unambiguously identified by unique peptides), and the number of spectral counts (MS/MS count) corresponding to each grouping are shown. The bait protein, HCE1, is highlighted in yellow. Protein IDs associated with human proteins are colored in blue. The gene product (for *Toxoplasma* proteins) or associated gene name (for human proteins) for the first listed protein ID in each row is shown in the Description column. Sheet 1 (“All_proteins”) shows the experimental data sets for all proteins, both human and *Toxoplasma*, listed in rank order by enrichment score (HCE1/RH, as further elaborated in Materials and Methods) in IP no. 1. Sheet 2 (“Human_proteins”) shows the experimental data sets for human proteins only listed in rank order by enrichment score in IP no. 1. Sheet 3 (“Toxo_proteins”) shows the experimental data for *Toxoplasma* proteins only listed in rank order by enrichment score in IP no. 1. Sheet 4 (“Parameters”) shows the parameters used in the MaxQuant analysis. Download Table S3, XLSX file, 0.1 MB. Copyright © 2019 Panas et al. 2019 Panas et al. This content is distributed under the terms of the Creative Commons Attribution 4.0 International license.

**FIG 6:**
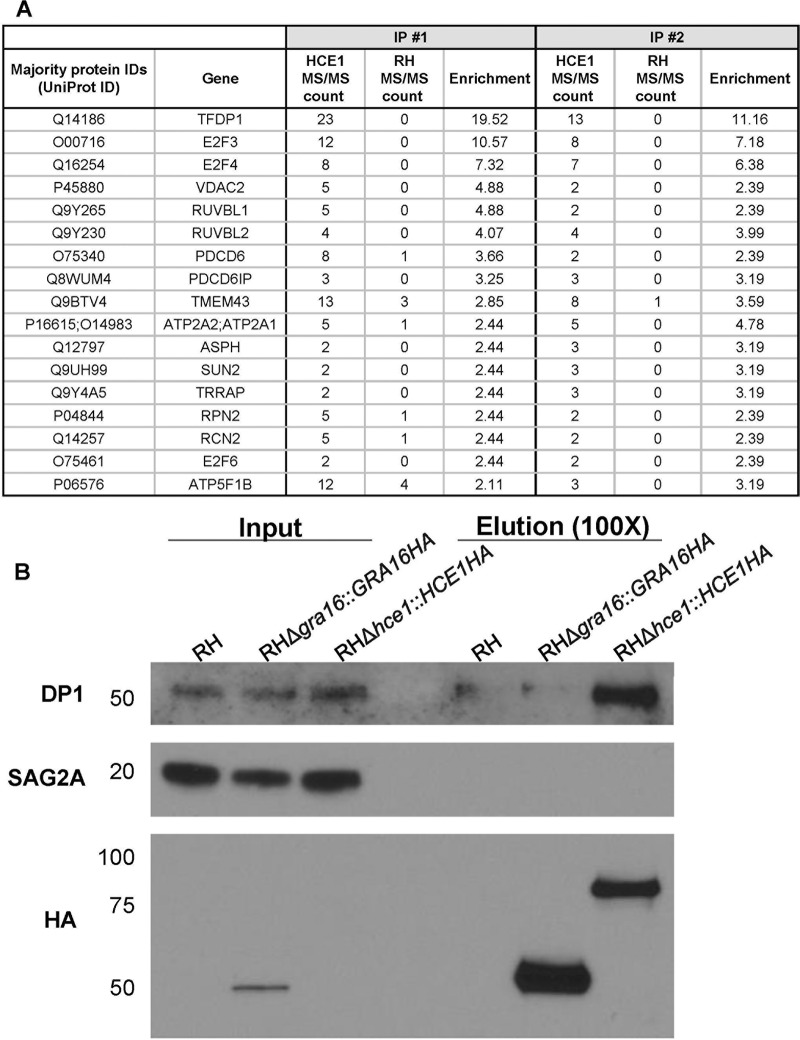
HCE1 specifically associates with host TFDP1 and E2F. (A) Anti-HA magnetic beads were used to immunoprecipitate proteins from HFFs infected for 24 h with the RH wild-type strain or mutant RHΔ*hce1*::*HCE1HA*. Mass spectrometry was performed on the resulting material, and the number of spectral counts was determined for all detectable proteins (IP no. 1). The experiment was then repeated under the same conditions except that the lysate was sonicated prior to immunoprecipitation to ensure release of all proteins that might be trapped in membranous material (IP no. 2). The results from the two experiments were ranked according to the enrichment of spectral counts in the HCE1-HA-expressing strain relative to the wild-type RH control after adding a nominal single count to all results, thereby enabling a ratio to be determined, and after accounting for the total spectral counts identified in each experiment (Enrichment). Given that much of HCE1 within an infected cell is inside the dense granules and/or PV space and thus can associate with other GRA proteins, possibly nonspecifically, we used a higher threshold for enrichment for parasite proteins to be included in this abbreviated list. The full data set is presented in Table S3. Displayed are the majority protein identifiers (IDs), i.e., those corresponding to the proteins which contain at least half of the peptides belonging to a protein group (i.e., a grouping of proteins which cannot be unambiguously identified by unique peptides), the corresponding gene for those proteins, and the corresponding number of spectral counts (MS/MS count) for all human proteins with an enrichment score greater than 2 in both of the experiments. The three most highly enriched proteins in both experiments were DP1, E2F3, and E2F4. (B) Lysates from a repeat of IP no. 2, including an additional control group consisting of parasites expressing an unrelated, HA-tagged effector, GRA16HA (RH*Δgra16*::*GRA*16HA), were resolved by SDS-PAGE, blotted, and probed with antibodies to DP1, SAG2A (as a specificity and parasite input control), and anti-HA (to show efficient immunoprecipitation of the relevant HA-tagged protein). Approximately 100-fold-more starting material was represented in the eluate than in the input—hence the stronger bands corresponding to the HA-tagged material in the eluate than in the input. DP1 was specifically enriched in the immunoprecipitation from the HCE1HA-expressing parasites. The blot represents a single replicate.

When the immunoprecipitation data were examined for human proteins that appeared to specifically associate with HCE1, we observed 17 with enrichment scores greater than 2 in both experiments (Fig. 6A; a complete list is given in Table S3). The three proteins that were most strongly enriched were DP1, E2F3, and E2F4, which were identified with at least 7 spectral counts (and at least 6 unique peptides) in both immunoprecipitations with HCE1HA and 0 spectral counts in both immunoprecipitations with the negative control, RH. Note that E2F3 was expressed as two variants differing in the initiating methionine; E2F3a has an N-terminal extension of approximately 120 amino acids over E2Fb, and when we mapped unique peptide fragments to these two isoforms, we found that 12 of 13 fragments mapped to the common region but that 1 peptide fragment, with 1 spectral count in each IP, mapped to the region unique to E2F3a (Fig. S3). Thus, while the data are unambiguous in indicating the presence of E2F3, we cannot assert whether E2F3b was present or absent but would argue that the E2F3a variant, at least, was present.

10.1128/mBio.00674-19.3 FIG S3 Alignment of E2F3a and E2F3b and identification of peptide fragments that immunoprecipitated with HCE1. Peptide fragments 1 to 13 are displayed in a table (A), and the data were mapped to an alignment of E2F3a and E2F3b (B). Blue lines represent the peptide fragment, and the fragment number is indicated immediately to the right of the blue line. Only peptide fragment 1, for which there was one spectral count that was found to associate with HCE1 in each IP, mapped to a region unique to E2F3a. All other peptide fragments mapped to regions common to E2F3a and E2F3b. Download FIG S3, DOCX file, 0.5 MB. Copyright © 2019 Panas et al. 2019 Panas et al. This content is distributed under the terms of the Creative Commons Attribution 4.0 International license.

Other components of the DP1-E2F transcription complex were also observed in the HCE1HA-associating material, including E2F6, TRRAP (a histone acetyltransferase that associates with DP1-E2F), and RUVB1/RUVB2 (members of the AAA-positive [AAA^+^] family of ATPases associated with various cellular activities and reported binders of E2F1) (19, 20). Six of the remaining 10 proteins in the list had one or more spectral counts in the negative control, making their specific association with HCE1 questionable. To validate the association of HCE1 with the E2F/DP1 transcription complex, we focused on DP1, which is the stably associated dimerization partner (hence “DP1”) of almost all E2Fs. For this validation, we repeated the infection and immunoprecipitations and included parasites expressing GRA16HA as an additional specificity control, since this protein is also present in the infected host cell nucleus (9). We then probed the eluates of the immunoprecipitation with an anti-DP1 antibody and with antibodies to SAG2A (as a loading control for input material) and HA (to verify that the immunoprecipitation had been successful; Fig. 6B). Although expression of HCE1HA was not detected in the input material, following immunoprecipitation and enrichment with anti-HA, HCE1HA was observed to migrate at ∼80 kDa, consistent with its predicted size of 68.4 kDa (with HA tag and after removal of signal peptide) and allowing for the fact that it is phosphorylated at a minimum of 4 positions (Fig. 1A) (16). Importantly, the results showed that DP1 is substantially enriched in the material coprecipitating with HCE1HA relative to the wild-type and GRA16HA-expressing negative controls, confirming the specific association of DP1 with HCE1HA. Although we cannot discern from these results whether this association is direct or indirect (e.g., HCE1HA binds to E2F3 and E2F4 and, thereby, to DP1), they do provide the beginning of a mechanistic explanation for how HCE1 regulates cyclin E, as discussed further below.

### HCE1 is sufficient to upregulate cyclin E during infection with Neospora caninum and under conditions of expression in uninfected HFFs.

**FIG 7:**
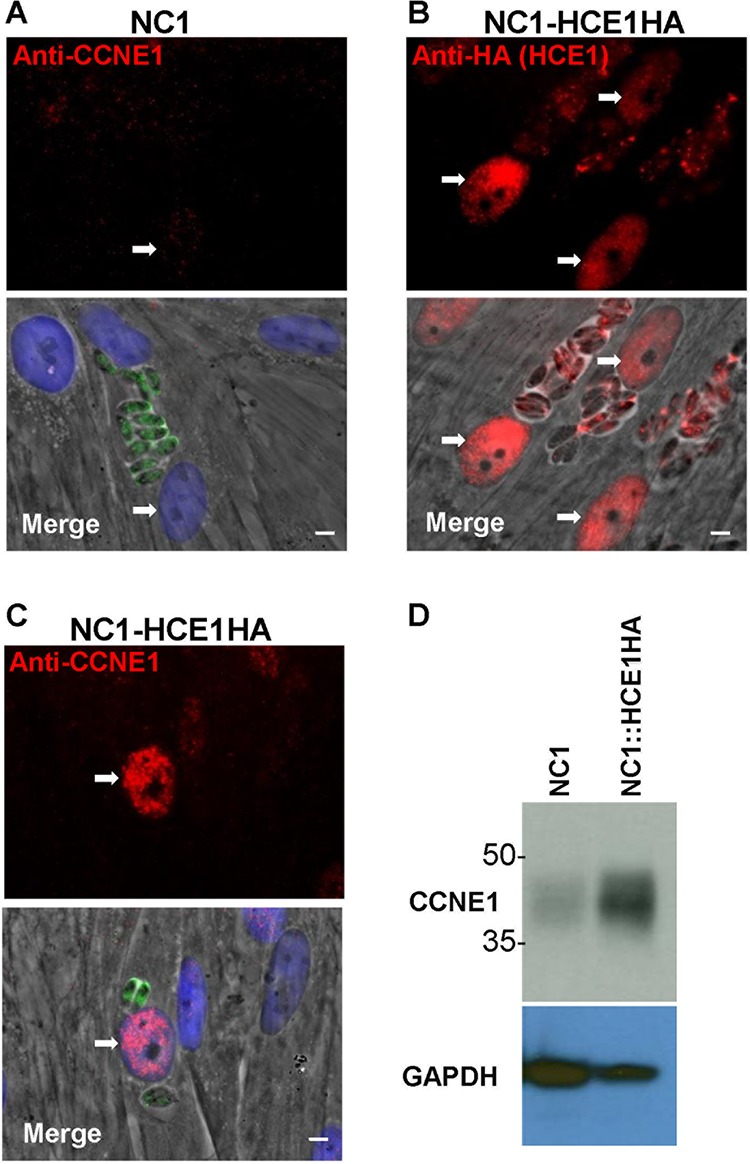
Expression of HCE1 in *Neospora* allows it to control host cyclin E1. (A) IFA with anti-CCNE1 of HFFs infected with wild-type Neospora caninum NC1 showing no CCNE1 induction in the nucleus of the infected host cell (white arrows). The white scale bar represents 5 μm. (B) The experiment was performed as described for panel A except the IFA was performed with anti-HA of HFFs infected with NC1 stably transfected with *pGRA-TGGT1_239010HA*, showing that the transfected NC1 expressed, exported, and localized HCE1 to the infected cell’s nucleus. (C) The experiment was performed as described for panel B except that the IFA was performed with anti-CCNE1, showing efficient CCNE1 induction in the nuclei of the infected host cell. (D) Lysates of HFFs infected with the indicated NC1 strain for 20 h were resolved by SDS-PAGE, blotted, and probed with antibodies to CCNE1 or GAPDH (as a loading control). The blot is representative of results from three independent experiments.

Given the evidence from previous transcriptomic experiments with bovine trophoblast cells (14), plus the extreme divergence between HCE1 and the *Neospora* orthologue BN1204_015825 reported by NCBI BLASTP, we hypothesized that Neospora caninum would not induce cyclin E1 in human cells. This was tested and confirmed to be the case (Fig. 7A). To ask if HCE1 would be sufficient to confer the ability to upregulate cyclin E1 to its Apicomplexan cousin, we first generated a HCE1-expressing strain of *Neospora* and tested if the introduced protein would be exported to the host nucleus. As shown in Fig. 7B, the HCE1 was indeed translocated to the host nucleus, and so we then asked if the resulting strain would induce cyclin E. The results (Fig. 7C) showed that HFFs infected with this engineered strain showed strong upregulation of cyclin E. Western blotting (Fig. 7D) confirmed this finding on the population level. Collectively, these results indicate that *Neospora* has the ability to produce, process, and export a fully functional HCE1 and that HCE1’s activity is not dependent on other effectors specific to *Toxoplasma*.

**FIG 8:**
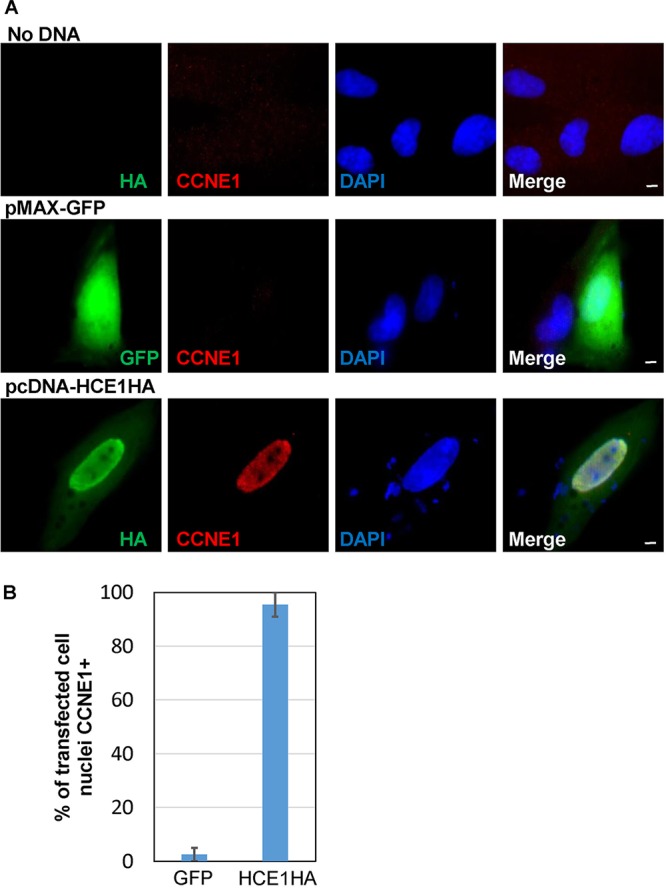
Expression of HCE1 in HFFs, without infection, is sufficient to upregulate cyclin E1 at 20 h. (A) HFFs were subjected to mock transfection with no DNA or were transfected with human expression plasmid pMAX-GFP or with pcDNA-HCE1HA, as indicated, and then assessed by IFA for CCNE1 expression at 20 h. The white scale bar represents 5 μm. (B) Quantification of the HFF cultures shown in panel A, showing the mean percentages of host nuclei in cells expressing the indicated transgene that were positive for CCNE1. Data shown represent averages of results from assessments of two independent experiments, and error bars indicate standard errors of the means.

We next tested whether HCE1 is sufficient to induce cyclin E on its own, with no infection and no other parasite proteins present. To do this, we cloned a truncated version of the HCE1 open reading frame, lacking the predicted signal sequence for export, into the human expression vector pcDNA under the control of the cytomegalovirus (CMV) promoter and used Lipofectamine LTX to transfect this into subconfluent HFFs. Subconfluent HFFs were utilized in this experiment because Lipofectamine LTX shows higher transfection efficiency than confluent HFFs. Neither the transfection procedure alone (“No DNA”) nor expression of an irrelevant gene (a green fluorescent protein [GFP] gene) under the control of the CMV promoter induced robust cyclin E1 (Fig. 8A), although a small fraction (∼2.5%) of cells did express detectable cyclin E1 as expected for these subconfluent HFF cultures containing replicating cells (Fig. 8B). Transfection of the plasmid pcDNA-HCE1HA, however, resulted in strong expression of HCE1, a majority of which was concentrated within the cell nucleus (Fig. 8A). Importantly, >90% of cells expressing the HCE1 transgene exhibited this robust level of cyclin E1 induction (Fig. 8B).

## DISCUSSION

We showed here that HCE1 is an effector protein that is both necessary and sufficient for robust upregulation of cyclin E in the host, independently of all other parasite proteins. Mechanistically, once HCE1 arrives in the host cell nucleus, it appears to function via a direct interaction with E2F/DP1 heterodimers, most prominently in the confluent HFFs studied here, i.e., those comprised of E2F3 and E2F4. We also detected binding to E2F6, to the E2F-associating histone acetyltransferase TRRAP (20), and to two AAA^+^ proteins that participate in chromatin remodeling, namely, RUVB1 and RUVB2 (19). When DP1 is bound to E2F3, the complex is considered nominally “activating,” while when bound to E2F4 or E2F6 the complex is generally considered “inhibitory” (21, 22); however, these characterizations depend heavily on other members of the transcription complex. For example, when the cells are in the resting G_0_ state or the early G_1_ phase, before commitment to entry into the cell cycle, the members of the retinoblastoma (RB) family of proteins (RB1, RBL1, RBL2) associate with E2F/DP1 dimers to maintain repression at the promoters of cyclin E and other E2F targets. Without RBL1 or RBL2, E2F4 has the potential to function as an activating transcription complex (23). Unlike what has been reported for adenovirus E1A, human papillomavirus 16 (HPV-16) E7 proteins, and the large T antigen of polyomaviruses, which can all bind RB to release active E2F (24–26), we did not observe binding of HCE1 with any of the members of the RB family of proteins. Collectively, our results instead suggest a model in which HCE1 retains DP1/E2F3 and DP1/E2F4 in an active state, tied to the coactivator TRRAP, and without the suppressive effects of the RB pocket proteins.

Several publications have described the active interference of *Toxoplasma* tachyzoites with host cell cycle machinery (1, 2, 27, 28), including interference operating in MYR1-dependent ways (12). The results presented here for HCE1 likely provide at least a partial explanation for these effects, since, among the E2F-regulated genes whose expression is HCE1 dependent, we saw several genes related to the prereplication complex such as the minichromosome maintenance (MCM) genes and DNA polymerase E and origin replication complex subunit 1 (ORC1) genes. Previous observations revealing that *Toxoplasma*-infected host cells were induced from G_1_ into S and yet halted at the G_2_/M boundary have suggested that multiple effectors, i.e., both an activator of the cell cycle and, later, an inhibitor, were likely in play. GRA16 is known to impact p53 (9), GRA24 impacts p38 mitogen-activated protein (MAP) kinase signaling (29), and both affect the cell cycle. In the case of GRA16, the presence of a strong p53 signature, combined with data demonstrating a decrease in cyclin B levels, is consistent with its being one of the effectors responsible for stalling the cell cycle in G_2_ (9). This may cause a halt to the effect that is launched by HCE1, preventing a cell from actually dividing. Determining exactly how each effector’s function interfaces with the others in controlling cell cycle and what specific benefit this disruption provides to the growth of tachyzoites (evident from the fact that *Δhce1* parasites produce significantly smaller plaques than wild-type parasites) will require substantial further study. It may well be that launching a cell into an abortive process of growth and division provides a wealth of nutrients for the parasites to use for their own growth, and metabolomic studies of wild-type-infected and strain *Δhce1*-infected cells will help reveal whether such might be occurring. Regardless, the finding here that HCE1 is the protein responsible for cyclin E upregulation reveals its role as a critical lynchpin in the process by which *Toxoplasma* tachyzoites so capably co-opt infected host cells for their own purposes.

## MATERIALS AND METHODS

### Parasite strains, culture, and infections.

The following strains were used in this study: *Toxoplasma* RHΔ*hxgprt* (2), RHΔ*myr1* (2), RHΔ*asp5* (30), RHΔ*gra16* (6), RHΔ*gra16*::*GRA16HA* (6), and Neospora caninum NC-1 (31). *Toxoplasma* and *Neospora* tachyzoites were propagated in human foreskin fibroblasts (HFFs) cultured in complete Dulbecco’s modified Eagle medium (cDMEM) supplemented with 10% heat-inactivated fetal bovine serum (FBS; HyClone, Logan, UT), 2 mM l-glutamine, 100 U ml^−1^ penicillin, and 100 μg ml^−1^ streptomycin at 37°C with 5% CO_2_. These strains may be obtained by contacting us.

The HFFs were obtained from the neonatal clinic at Stanford University following routine circumcisions that were performed at the request of the parents for cultural, health, or other personal medical reasons (i.e., for reasons not in any way related to research). These foreskins, which would otherwise have been discarded, were fully deidentified and therefore do not constitute “human subject research.” Prior to infection, parasites were scraped and lysed using a 27-gauge needle, counted using a hemocytometer, and added to HFFs at the stated multiplicity of infection. “Mock” infection was done by first subjecting uninfected HFFs to syringe lysing, processing this in the same manner as was done for the infected cells, and then adding the same volume of the resulting material as was used for the infections.

### Transfections.

All transfections were performed using a *BTX* EMC600 electroporation system (Harvard Apparatus). Tachyzoites were mechanically released in phosphate-buffered saline (PBS), pelleted, and resuspended in solution for transfection. After transfection, parasites were allowed to infect HFFs in DMEM. Transfections were performed using 5 × 10^6^ to 10 × 10^6^ parasites and 3 to 10 μg DNA in CytoMix (10 mM KPO_4_ [pH 7.6], 120 mM KCl, 5 mM MgCl_2_, 25 mM HEPES, 2 mM EDTA, 150 μM CaCl_2_).

### Immunofluorescence assay (IFA).

Infected cells grown on glass coverslips were fixed using 4% formaldehyde–PBS for 20 min. Samples were washed once with PBS and blocked using 3% bovine serum albumin (BSA)–PBS for 1 h at room temperature (RT). Cells were permeabilized with 0.2% Triton X-100–3% BSA–PBS for 10 min at RT. Cyclin E was detected with mouse monoclonal antibody HE111 (Santa Cruz Biotechnology), GRA7 was detected with rabbit polyclonal anti-GRA7 serum, and HA was detected with rat monoclonal antibody 3F10 (Roche). Secondary anti-mouse, anti-rat, and anti-rabbit antibodies were used conjugated to Alexa Fluor 488 and 594. Vectashield was used with DAPI (4′,6-diamidino-2-phenylindole) stain (Vector Laboratories) to mount the coverslips on slides. Fluorescence was detected using wide-field epifluorescence microscopy, and images were analyzed using ImageJ or Fiji. All images shown for any given condition/staining in any given comparison/data set were obtained using identical parameters.

### Gene disruption.

The RHΔ*HCE1* and RHΔ*gra16* strains were generated by disrupting the corresponding gene loci using CRISPR-Cas9 and selecting for integration of a vector encoding hypoxanthine-guanine phosphoribosyltransferase (*HXGPRT*) using drug selection. Specifically, the pSAG1:U6-Cas9:sgUPRT vector (32) was modified by Q5 site-directed mutagenesis (NEB) to specify a single guide RNA (sgRNA) targeting *TGGT1_239010*. The resulting sgRNA plasmid, dubbed pSAG1:U6-Cas9:sg239, was transfected into strain RHΔ*hpt* along with the previously described pTKO2 vector (33), which carries the *HXGPRT* gene flanked by *loxP* sites. Parasites were allowed to infect HFFs for 24 h, after which time the medium was changed to cDMEM supplemented with 50 μg/ml mycophenolic acid (MPA) and 50 μg/ml xanthine (XAN) for HXGPRT selection. The parasites were subjected to two passages before being singly cloned into 96-well plates by limiting dilution. Disruption of the gene-coding regions was confirmed by PCR using primers 239010F (GCACGAACCATAGAAAAGTAGGAA) and 239010R (AGTGGTCGCTGGCGTGCTGTT) and then sequencing of the amplification products.

### Complementation.

The RHΔ*239010* strain (also referred to as strain RH*Δhce1*) was complemented ectopically with the pGRA-239010-HA (pGRA-HCE1HA) plasmid. Expression of *TGGT1_239010*/*HCE1* is driven by its natural promoter. To construct the pGRA-239010-HA plasmid, the *TGGT1_239010* promoter and open reading frame were amplified from strain RHΔ*hpt* genomic DNA using ACTAAAGCTTTTAGGCCAAAAACTGCACCCATCC and TAGTTTAATTAACTACGCGTAGTCCGGGACGTCGTACGGGTAGGAAGATCCGTCCGACATTCTTC primers. The resulting ∼3.5-kb fragment was digested with HindIII and PacI restriction enzymes and cloned into the corresponding sites of the pGRA1 backbone. Five micrograms of the resulting vector, pGRA-HCE1-HA, was cotransfected with 3 μg pSAG1:U6-Cas9:sgUPRT (32) into strain RHΔ*hce1* tachyzoites to create strain RHΔ*hce1*::*HCE1-HA*. Integration of the vector at the *UPRT* locus was enriched by selecting for resistance to 5 μM FUDR in DMEM after one lytic cycle. The resulting population was then cloned by limiting dilution and tested for HCE-HA expression by IFA.

### Gene expression in human foreskin fibroblasts.

The *239010* open reading frame was cloned from genomic DNA isolated from RH-infected HFF. Primers TAGTGCGGCCGCATGTTTGCAAGCGCCGGAACGGG and TAGTCTCGAGCTACGCGTAGTCCGGGACGTCGTACGGGTAGGAAGATCCGTCCGACATTCTTC were used for amplification of the reading frame after the signal sequence (beginning with the codons encoding PheAlaSerAla), addition of a C-terminal HA tag, and insertion into pcDNA after digestion with NotI and XhoI. GFP was expressed from the pMAX-GFP plasmid. DNA (1,000 to 1,500 ng) was transfected into subconfluent HFFs using Lipofectamine LTX (Thermo) according to the manufacturer’s protocol.

### Western blotting.

Infections were performed for Western blotting at an MOI of 2:1. Parasites were subjected to syringe lysing using a 27-gauge needle and counted, and equivalent numbers of parasites were used for infections. Cell lysates were harvested 20 h postinfection (hpi) and suspended in radioimmunoprecipitation assay (RIPA) buffer with protease and phosphatase inhibitors (Roche, Thermo Fisher). Samples containing 10 to 20 μg protein were boiled for 20 min in sample buffer, separated by SDS-PAGE, and transferred to polyvinylidene difluoride (PVDF) membranes. Membranes were blocked in 5% milk–Tris-buffered saline (TBS) supplemented with 0.2% Tween 20 (TBST) for 1 h at RT and then incubated for 1 h at RT with primary antibody in blocking buffer. Cyclin E1 was detected using mouse monoclonal antibody HE12 (Santa Cruz Biotechnology) and goat anti-mouse secondary antibody conjugated to horseradish peroxidase (HRP). GAPDH (glyceraldehyde-3-phosphate dehydrogenase) was detected with mouse monoclonal antibody 6C5 (Calbiochem). SAG1 levels were used to control for the levels of parasites within the infected cells, and blots were stained with polyclonal rabbit anti-SAG1 followed by a secondary goat anti-rabbit antibody conjugated to HRP. The HA epitope was detected using horseradish peroxidase (HRP)-conjugated HA antibody (Roche; catalog no. 12013819001 ab 3F10). SAG2 and DP1 were recognized by rabbit polyclonal anti-SAG2 (generated previously [34]) and mouse monoclonal anti-DP1 (Santa Cruz Biotechnology, catalog no. sc-70990), respectively. For anti-DP1, a secondary antibody that recognizes the nonreduced form of mouse IgG (Abcam catalog no. ab131368) was used. HRP was detected using an enhanced chemiluminescence (ECL) kit (Pierce). Membranes were stripped between blots by incubation in stripping buffer (Thermo Fisher) for 10 tot 15 min and were then washed twice for 5 min each time with TBST.

### Plaque assay.

HFFs were grown to confluence in a T25 flask. A total of 200 parasites were added to each T25 flask and incubated for 7 days. The infected monolayers were washed with PBS, fixed using methanol, and stained with crystal violet. Plaque sizes were measured using ImageJ.

### RNA extraction, library preparation, and sequencing.

HFFs were subjected to serum starvation for 24 h before infection after growth in DMEM containing 0.5% serum. They were then infected with the indicated line of tachyzoites at an MOI of 5, and at 6 hpi, 1 ml TRIzol reagent (Invitrogen) was added to each T25 flask and the cells were scraped. Lysates were collected into RNase/DNase-free Eppendorf tubes and frozen at −20°C. Total RNA was extracted following the manufacturer’s instructions, with some modifications. Briefly, frozen samples were thawed on ice and 0.2 ml chloroform was added to TRIzol suspensions, which were then mixed by inversions performed 10 times and incubated for 5 min. Tubes were then spun at 12,000 rpm for 15 min at 4°C. RNA in the aqueous phase was transferred into a fresh tube, and 0.5 ml absolute isopropyl alcohol was added. Each tube was inverted three times and incubated at 4°C for 10 min. The tubes were then spun at 12,000 rpm for 20 min at 4°C. After the supernatants were decanted, the RNA pellets were washed with 1 ml 75% ethanol. The tubes were mixed by inverting the tubes 10 times and were then spun at 12,000 rpm for 20 min at 4°C. Supernatants were removed, and the RNA pellets were air-dried in open tubes for approximately 10 min. The RNA pellets were resuspended in 30 μl RNase-free diethyl pyrocarbonate (DEPC)–water. Multiplex sequencing libraries were generated using an RNA sample preparation kit (Illumina), and the samples were submitted to the Stanford University Functional Genomic Facility (SFGF) for purity analysis using an Agilent 2100 Bioanalyzer. The samples, having been barcoded to preserve identity, were then pooled for a single high-throughput sequencing run using an Illumina NextSeq platform (Illumina NextSeq 500). Infection and harvest were done twice independently.

### RNA-Seq read mapping and differential expression analysis.

Raw reads were uploaded onto the CLC Genomics Workbench 8.0 (Qiagen) platform for independent alignments against the human genomes (Ensembl.org/hg19). All parameters were left at their default values. The number of total reads mapped to each genome was used to determine the number of RPKM (reads per kilobase of transcript per million mapped reads). Heat maps were generated using Gene E (https://software.broadinstitute.org/GENE-E/index.html).

### Gene set enrichment analysis (GSEA).

GSEA, available through the Broad Institute at http://www.broadinstitute.org/gsea/index.jsp, was the enrichment analysis software used to determine whether the defined sets of differentially expressed human genes analyzed in our experiment showed statistically significant overlap of gene sets in the curated Molecular Signatures Databases (MsigDB) Hallmark gene set collection. We used the cutoff of *P* values of ≤10^−4^. The list of genes that are found in the gene sets presented is provided in Table S2.

### Immunoprecipitations (IPs) for mass spectrometry samples.

IPs to identify HCE1-interacting proteins in HFFs were performed as follows. One 15-cm-diameter dish of HFFs was grown to confluence for each infection condition. HFFs were infected with 15 × 10^6^ RH, RHΔ*hce1*::*HCE1HA*, or RHΔ*gra16*::*GRA16HA* parasites for 24 h. Infected cells were washed 3 times in cold PBS and then scraped into 1 ml cold cell lysis buffer (50 mM Tris [pH 8.0], 150 mM NaCl, 0.1% [vol/vol] Nonidet P-40 Alternative [CAS no. 9016-45-9]) supplemented with complete protease inhibitor cocktail (cOmplete; EDTA-free [Roche]). The cell lysate was passed 3 times through a 25-gauge needle, followed by passage 3 times through a 27-gauge needle to break up the cells. For IP no. 2 only, the cell lysate was then subjected to sonication on ice (Branson Sonifier 250, with 3 pulses of 10 s at 50% duty cycle and output control 2). Cell lysates were spun at 1,000 × *g* for 10 min to remove insoluble material and unlysed cells. Lysates were added to 100 μl magnetic beads conjugated to anti-HA antibodies (Pierce) and incubated overnight with rotating at 4°C. Unbound protein lysate was removed, and the anti-HA magnetic beads were then washed 10 times in cell lysis buffer. HA-tagged proteins were eluted in 60 μl pH 2.0 buffer (Pierce) for 10 min at 50°C to dissociate proteins from the antibody-conjugated beads. The elutions were immediately neutralized at a 1:10 dilution with pH 8.5 neutralization buffer (Pierce).

### Mass spectrometry sample preparation.

A 45-μl volume of each IP elution was combined with 15 μl of 4× Laemmli sample buffer supplemented with BME (2-mercaptoethanol) (Bio-Rad), boiled for 10 min at 95°C, and loaded on a Bolt 4%-to-12% Bis-Tris gel (Invitrogen). The samples were resolved for approximately 8 min at 150 V. The gel was washed once in UltraPure water (Thermo) and fixed in 50% methanol–7% acetic acid for 15 min, followed by 3 additional washes with UltraPure water. The gel was stained for 10 min with GelCode Blue (Thermo) and washed with UltraPure water for an additional 20 min. One gel band (approximately 1.5 cm in size) for each condition was excised and destained for 2 h in a 50% methanol and 10% acetic acid solution, followed by a 30-min soak in UltraPure water. Each gel slice was cut into 1-mm-by-1-mm squares, covered in 1% acetic acid solution, and stored at 4°C until the in-gel digestion could be performed.

To prepare samples for mass spectrometry, the 1% acetic acid solution was removed, 10 μl of 50 mM dithiothreitol (DTT) was added, and the volume was increased to 100 μl with 50 mM ammonium bicarbonate. Samples were incubated at 55°C for 30 min. The samples were then cooled to RT, the DTT solution was removed, 10 μl of 100 mM acrylamide (propionamide) was added, and the volume was again normalized to 100 μl with 50 mM ammonium bicarbonate followed by an incubation at RT for 30 min. The acrylamide solution was removed, 10 μl (0.125 μg) of trypsin/LysC (Promega) solution was added, and another 50 μl of 50 mM ammonium bicarbonate was added to cover the gel pieces. Samples were incubated overnight at 37°C for peptide digestion. Solution consisting of digested peptides was collected in fresh Eppendorf tubes, and 50 μl of extraction buffer (70% acetonitrile, 29% water, 1% formic acid) was added to gel pieces, incubated at 37°C for 10 min, centrifuged at 10,000 × *g* for 2 min, and collected in the same tubes consisting of the previous elute. This extraction was repeated one more time. Collected extracted peptides were dried to completion in a SpeedVac and stored at 4°C until ready for mass spectrometry.

### Mass spectrometry.

Eluted, dried peptides were dissolved in 12.5 μl of 2% acetonitrile–0.1% formic acid, and 3 μl was injected into an in-house-packed C_18_ reverse-phase analytical column (15 cm in length). Peptides were separated using a Waters M-Class ultraperformance liquid chromatography (UPLC) system, operated at 450 nl/min using a linear 80-min gradient from 4% mobile phase B to 40% mobile phase B. Mobile phase A consisted of 0.2% formic acid–99.8% water, and mobile phase B was 0.2% formic acid–99.8% acetonitrile. Ions were detected using an Orbitrap Fusion mass spectrometer operating in a data-dependent fashion using typical “top speed” methodologies. Ions were selected for fragmentation based on the most intensely multiply charged precursor ions using collision-induced dissociation (CID). Data from these analyses were then transferred for analysis.

### Mass spectrometric analysis.

The .RAW data were searched using MaxQuant version 1.6.1.0 (35) against the canonical human database from UniProt, *Toxoplasma* GT1 databases from ToxoDB (versions 7.3 and 37.0), and the built-in contaminant database. Specific parameters used in the MaxQuant analysis can be found in Table S3. Peptide and protein identifications were filtered using a 1% false-discovery rate (FDR), and reversed proteins, contaminants, and proteins identified by only a single modification site were removed from the data set. HCE1HA enrichment over the non-HA-tagged RH was determined by adding a value of 1 to each spectral count (tandem MS [MS/MS] count), calculating the relative spectral counts observed in each experiment (MS/MS counts for each protein/total MS/MS counts for all proteins in that experiment), and then calculating the relative spectral counts observed for each protein in the two samples.

### Statistical analyses.

Statistical analysis was performed with Prism version 8 software. Analysis of plaque size was performed using Student's *t* test.

### Data availability.

The RNA-Seq data files have been deposited in GEO under accession number GSE122786. The mass spectrometry proteomics data have been deposited into the ProteomeXchange Consortium (http://proteomecentral.proteomexchange.org) via the PRIDE partner repository (36) with the data set identifier PXD012103.
