# Supplementary figures and images for: Toxoplasma Controls Host Cyclin E Expression through the Use of a Novel MYR1-Dependent Effector Protein, HCE1
Source: mBio. 2019 Apr 30;10(2):e00674-19. doi: 10.1128/mBio.00674-19 (PMC6495377; doi:10.1128/mBio.00674-19)

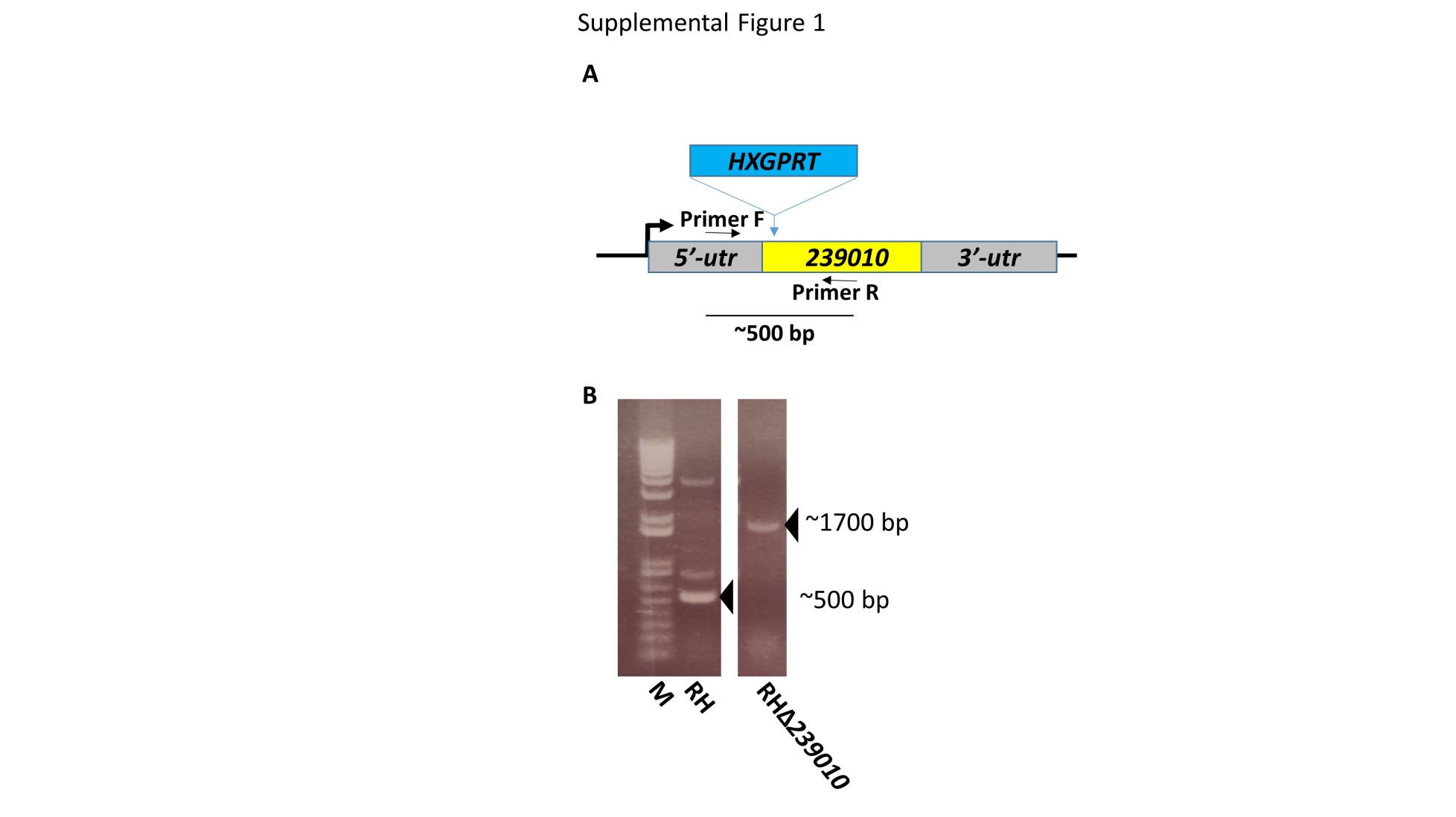

Supplement: FIG S1 [file mBio.00674-19-sf001.docx]

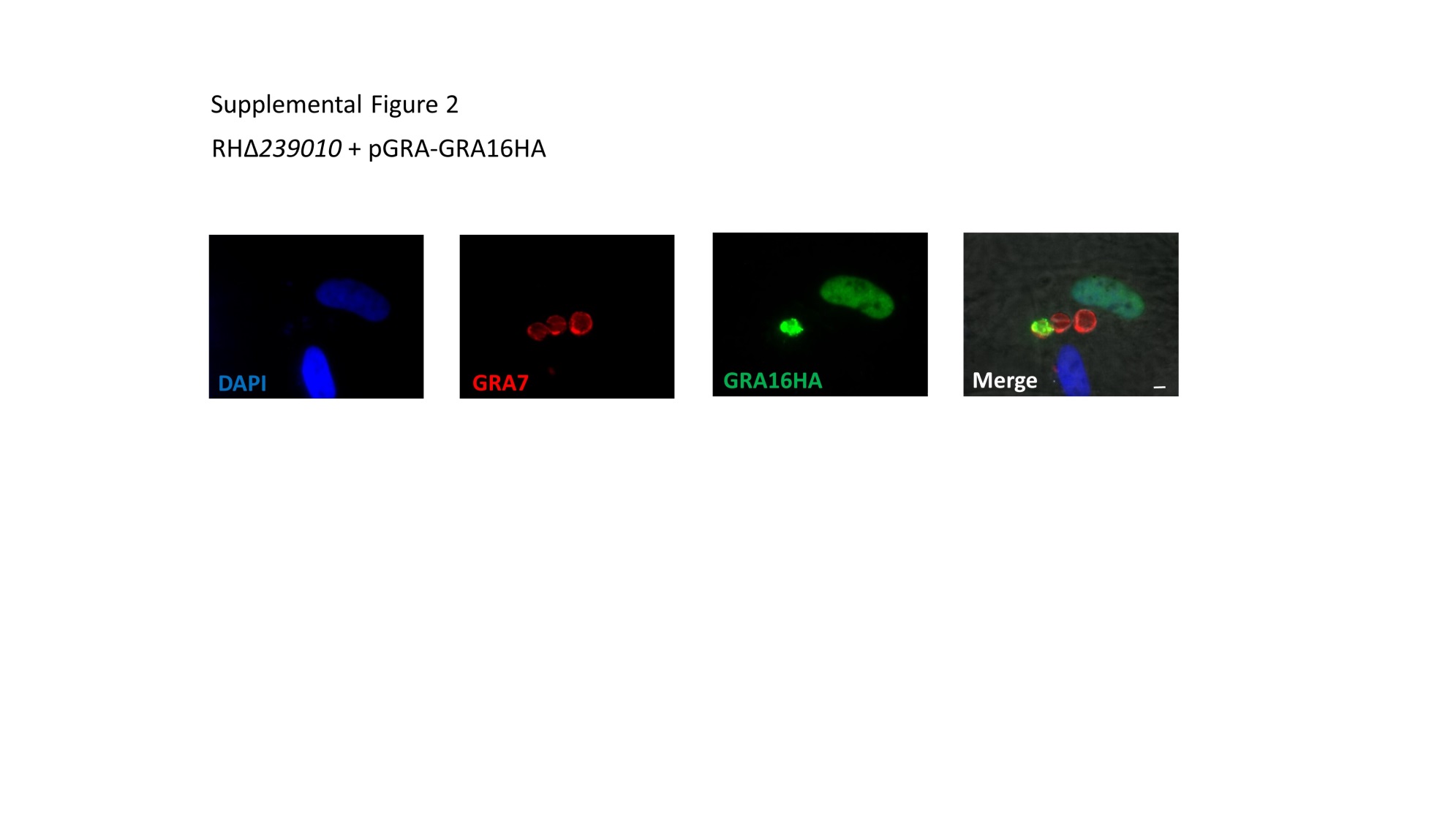

Supplement: FIG S2 [file mBio.00674-19-sf002.docx]

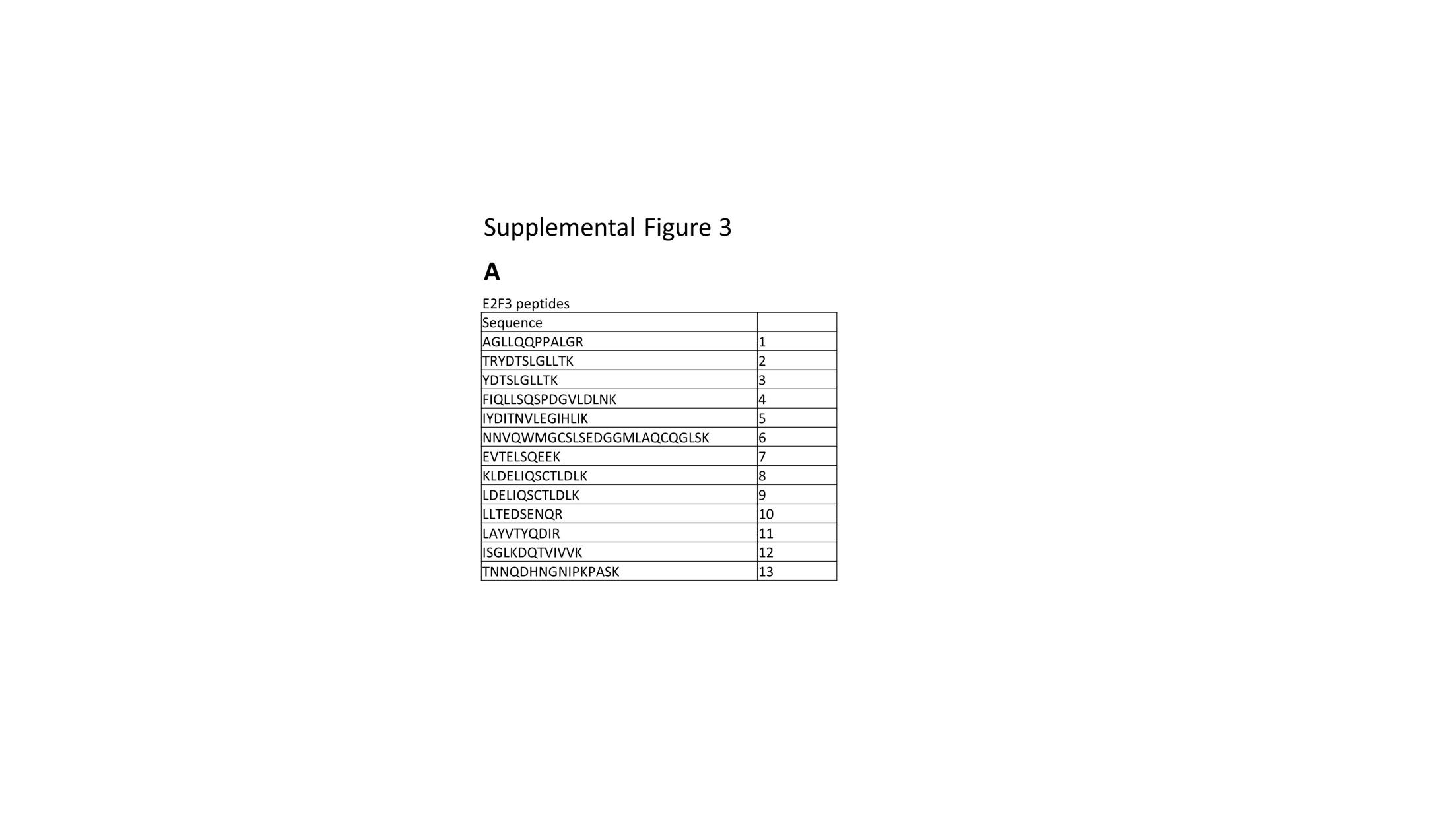


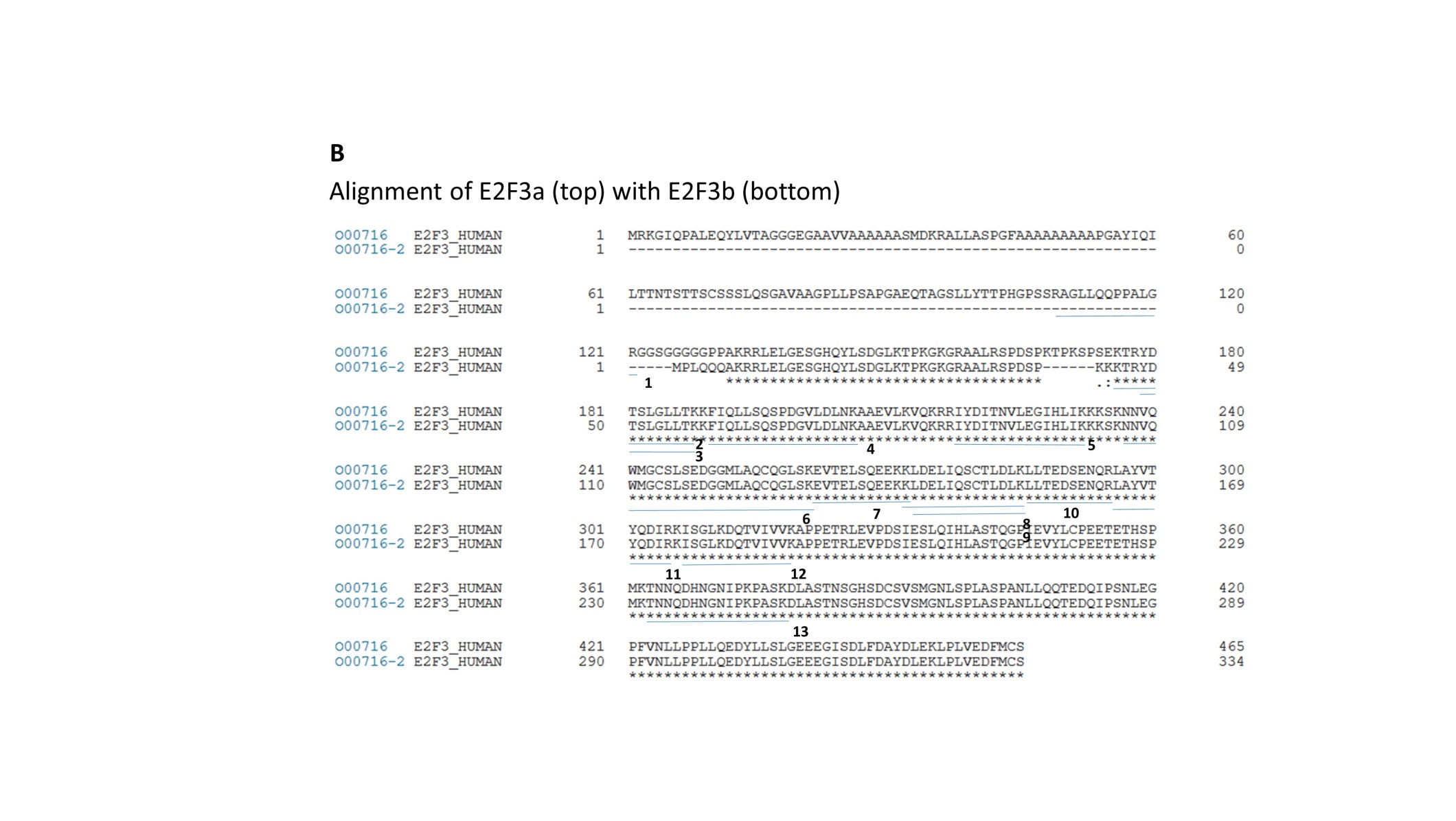

Supplement: FIG S3 [file mBio.00674-19-sf003.docx]
